# Impact of the 2019 European Guidelines on Diabetes in Clinical Practice: Real and Simulated Analyses of Lipid Goals

**DOI:** 10.3390/jcdd7010006

**Published:** 2020-02-05

**Authors:** Walter Masson, Melina Huerín, Lorenzo Martin Lobo, Gerardo Masson, Graciela Molinero, Mariano Nemec, Mariela Boccadoro, Cinthia Romero, Gabriel Micali, Daniel Siniawski

**Affiliations:** 1 Council of Epidemiology and Cardiovascular Prevention, Argentine Society of Cardiology, Buenos Aires C1115AAD, Argentina; walter.masson@hospitalitaliano.org.ar (W.M.); mhuerin@lezicacardio.com (M.H.); gmmassa@hotmail.com (G.M.); gracielamolinero@hotmail.com (G.M.); marianoanemec@hotmail.com (M.N.); lorenzomartinlobo@hotmail.com (M.B.); cinthiarromero@hotmail.com (C.R.); micalig@yahoo.com.ar (G.M.); drsiniawski@gmail.com (D.S.); 2 Argentine Society of Lipids, Córdoba X5000JGQ, Argentina

**Keywords:** diabetes, lipid goals, LDL-C, non-HDL-C

## Abstract

Background: Recent European guidelines on diabetes, prediabetes, and cardiovascular disease developed for the European Society of Cardiology (ESC) in collaboration with the European Association for the Study of Diabetes (EASD) significantly changed some concepts on risk stratification, lipid goals, and recommendations for the use of lipid-lowering drugs. The objectives of this work were to describe the lipid-lowering treatment prescribed for patients with diabetes and to determine the percentage of patients that achieved the lipid goals recommended by the 2019 ESC/EASD Guidelines on Diabetes in real and simulated scenarios. Methods: A multicenter, cross-sectional study was performed. Subjects >18 years with type 2 diabetes were included. The recommendations of the 2019 ESC/EASD Guidelines were followed. The real and simulated (ideal setting using adequate doses of statins ± ezetimibe) scenarios were analyzed. Results: Overall, 528 patients were included. In total, 62.5% of patients received statins (17.1% high intensity). Most patients were stratified as “very high risk” (54.2%) or “high risk” (43.4%). Only 13.3% achieved the double lipid goal (LDL-C and non-HDL-C goals according to the risk categories). In the simulation analysis, the proportion of subjects that did not reach the therapeutic objective decreased in all risk strata, although a considerable proportion of subjects persisted outside the target. Conclusion: The difficulty of achieving lipid goals in diabetic patients was considerable when applying the new guidelines. The situation would improve if we optimized treatment, but the prescription of new lipid-lowering drugs could be limited by their high cost.

## 1. Introduction

Consistent data have demonstrated the efficacy of statins in preventing cardiovascular events in patients with diabetes. A meta-analysis including patients with diabetes demonstrated that a statin-induced reduction of cholesterol bound to low-density lipoproteins (LDL-C) by 1.0 mmol/L was associated with a 9% reduction in all-cause mortality and a 21% reduction in the incidence of major cardiovascular events [1].

Scientific organizations issue management guidelines in order to influence everyday clinical practice professionals. Current guidelines recommend that diabetic patients should receive statins to lower LDL-C [2,3]. The choice of the type and dose of statin or the therapeutic objective to be reached varies according to the risk of the diabetic patient. However, management of dyslipidemia is suboptimal, even in very high risk patients. This is particularly observed for LDL-C, where a large majority of patients do not reach the treatment target [4,5].

Further intensification of LDL-C lowering occurs by adding nonstatin drugs with proven clinical efficacy. In the IMPROVE-IT trial, a significant reduction of the primary endpoint event rate in patients with diabetes and recent acute coronary syndrome receiving simvastatin and ezetimibe was reported, with a stronger beneficial effect on outcome than in patients without diabetes [6]. Likewise, a subgroup analysis of two landmark outcome trials with PCSK9 inhibitors demonstrated the efficacy of these drugs in the subpopulation with diabetes and coronary disease treated with statins [7,8].

Recent European guidelines on diabetes, prediabetes, and cardiovascular disease developed for the European Society of Cardiology (ESC) in collaboration with the European Association for the Study of Diabetes (EASD) significantly changed some concepts on risk stratification, lipid goals, and recommendations for the use of lipid-lowering drugs [9].

The objectives of our work were (a) to describe the lipid-lowering treatment prescribed for patients with diabetes, (b) to determine the percentage that achieved the lipid goals recommended by the 2019 European Guidelines on Diabetes, and (c) to analyze the proportion of patients who reached the lipid goals according to the same recommendations but according to a simulation model that estimated the reduction of lipids by adjusting the dose of statins at maximum intensity and adding ezetimibe for those who did not achieve the lipid goal.

## 2. Materials and Methods

A multicenter, descriptive, cross-sectional study was performed on consecutive samples obtained in the cardiovascular prevention outpatient clinics of six cardiology centers in the Autonomous City of Buenos Aires and Greater Buenos Aires. Subjects older than 18 years with diagnosis of type 2 diabetes were consecutively included, evaluating clinical and laboratory variables.

In this study, a detailed interview regarding risk profile was performed and all subjects underwent a clinical examination. Weight and height were registered and expressed as body mass index (weight in kilograms divided by height in meters squared). Waist circumference was measured in a horizontal plane, midway between the inferior margin of the ribs and the superior border of the iliac crest. Blood pressure was recorded using an automatic oscillometric blood pressure recorder after at least 5 min of rest on a chair and arm supported at heart level. The blood levels of glucose, total cholesterol, cholesterol bound to high-density lipoproteins (HDL-C), triglycerides, and creatinine were measured according to standardized biochemical tests. The LDL-C was calculated through Friedewald’s formula, while non-HDL-C was estimated by the following equation: total cholesterol − HDL-C.

To assess fulfillment of lipid goals and analyze the correct indication of statins, the 2019 ESC/EASD Guidelines on Diabetes were followed [9]. According to these recommendations, patients with diabetes were classified into three groups:

Very high risk: patients with diabetes and established cardiovascular disease, other target organ damage, or three or more major cardiovascular risk factors.

High risk: patients with diabetes duration ≥10 years without target organ damage plus any other additional risk factor.

Moderate risk: young patients (<50 years) with diabetes duration <10 years without other risk factors.

The LDL-C targets considered for the analysis were <100, <70, and <55 mg/dL for patients with moderate, high, and very high cardiovascular risk, respectively. On the other hand, the non-HDL-C goals considered for patients with very high, high, or moderate cardiovascular risk were <85, < 100, and <130 mg/dL, respectively.

A simulation model was carried out in order to estimate what the hypothetical decrease in LDL-C would be in an ideal setting using an intensive dose of statins (40/80 mg/day of atorvastatin or 20/40 mg/day of rosuvastatin) in patients who were receiving lower doses (the adjustment in the percentages of decrease in LDL-C varied depending on what the basal dose would have been in real conditions). Likewise, a decrease in LDL-C of 19% was estimated, simulating the theoretical effect of the addition of ezetimibe in those who did not receive it [10]. The non-HDL-C value was estimated by adding 30 mg/dL to the value obtained from LDL-C.

Continuous data were compared between groups, using the *t* test for normal distribution or the Mann–Whitney–Wilcoxon test for non-normal distribution. Continuous variables were expressed as mean ± standard deviation and categorical variables as percentages. A two-tailed *p*-value < 0.05 was considered statistically significant. STATA 11.1 and 3.1 EPIDAT software packages were used for statistical analysis.

The study was carried out following the medical research recommendations suggested by the Declaration of Helsinki, the Good Clinical Practice Guidelines, and current legal regulations.

## 3. Results

A total of 528 patients (mean age of 62.1 ± 12.7 years, 64.0% men) were included in the study. In total, 37.7% of the population was in secondary prevention (history of coronary heart disease, stroke, or peripheral vascular disease). Average values of total cholesterol, HDL-C, LDL-C, and non-HDL-C were 171.8 ± 43.4 mg/dL, 43.9 ± 12.4 mg/dL, 95.8 ± 38.5 mg/dL, and 127.9 ± 41.7 mg/dL, respectively. The median triglycerides were 139 mg/dL (interquartile range of 100–189 mg/dL). Importantly, 55.7% of the population was obese (body mass index ≥ 30 kg/m^2^) and 70.3% of patients were hypertensive. The characteristics of the population can be seen in Table 1.

In total, 62.5% of patients received statins, although only 17.1% were medicated with high-intensity statins. This proportion was higher in secondary prevention subjects compared with diabetics without cardiovascular history (23.4% vs. 12.7%; *p* = 0.04). The medication used in the population can be seen in Table 2.

Most patients were stratified as “very high risk” (54.2%) or “high risk” (43.4%). Only 13 subjects were classified as “moderate risk”. The proportion that achieved the lipid goals recommended by the 2019 European Guidelines on Diabetes according to the risk categories is described in Table 3.

In total, only 13.3% achieved the double lipid goal (LDL-C and non-HDL-C goals according to the risk categories). This proportion was higher in men than women (16.0% vs. 8.4%; *p* = 0.01). Similarly, a great proportion of subjects with a family history of early cardiovascular disease was observed in the group that achieved the double lipid goal (18.6% vs. 10.0%; *p* = 0.03). No significant differences were observed in the other variables evaluated between the groups with or without the double lipid goal achieved.

The use of statins, mainly those of high potency, was poor in our population. The statin schemes used in the different cardiovascular risk groups are shown in Table 4.

Also, a great proportion of subjects medicated with high-intensity statins was observed in the group that achieved the double lipid goal (28.6% vs. 14.6%; *p* = 0.003).

The simulation analysis contemplated an ideal scenario where everyone received the appropriate doses of statins, and if they did not reach the lipid target, ezetimibe was added. The proportion of subjects that reached the therapeutic goals increased in all risk strata (Table 5).

In total, 45.8% achieved the double lipid goal (LDL-C and non-HDL-C goals according to the risk categories) in the simulation analysis that assumed an adequate dose of statins in all patients. Likewise, the proportion increased to 56.4% when we simulated a clinical scenario where ezetimibe was added for patients who did not achieve the lipid goal.

## 4. Discussion

The main finding of our work was that many patients with diabetes did not achieve the lipid goals proposed by the new European guidelines. This was observed even in the simulated scenario where all patients were treated with statins with or without ezetimibe.

Dyslipidemia is one of the most common cardiovascular risk factors in patients with diabetes and is closely related to the risk of developing major cardiovascular outcomes [11]. Over the last few years, different guidelines have consistently recommended that lipid-lowering therapy intensity and lipid goals should be tailored according to cardiovascular risk profile. In our work, almost all of the patients were stratified with high or very high cardiovascular risk. Consequently, the intensity of the lipid-lowering treatment should be high.

Despite these recommendations, several observational studies reported poor control rates of LDL-C in this clinical setting [4,5,12,13,14]. This problem becomes more relevant if we consider that achievement of LDL-C goals was associated with better health outcomes among patients with diabetes [15].

The present work showed that diabetic women were less likely to be on optimal lipid-lowering therapy and consequently less likely to attain lipid goals compared to men. Although there is no recommendation that establishes differences between men and women, similar findings have been reported by other authors [16,17,18].

The reasons why patients with diabetes do not reach the recommended lipid objectives are manifold, highlighting the lack of adherence, medical inertia, and the poor response to statins. A large analysis of a secondary prevention database showed that in the population with diabetes, adherence reached only 55%, and the group of high-risk patients had the lowest levels of adherence [19]. On the other hand, therapeutic inertia, which is the lack of initiation or intensification of treatment by the physician when it is indicated, is common in diabetes care [20]. A Spanish study showed that in 639 patients with diabetes, medical inertia in terms of lipid control was 43.6% [21]. Finally, a study previously published in our country showed that diabetes was associated with a higher probability of obtaining a lower response to statins (hyporesponder subjects), probably related to the increase in intestinal cholesterol absorption observed in patients with type 2 diabetes [22].

Our findings showed a proportion of patients without adequate statin treatment like in other previously published reports. It is remarkable how this value remains at high levels over time, despite having robust evidence and clear recommendations from different scientific societies regarding their benefits. Likewise, the treatment deficit was also observed with nonstatin drugs. In fact, approximately 10% of our population received ezetimibe.

Recently, new guidelines for management of dyslipidemia were released by the ESC/EASD, proposing lower lipid goals than those recommended in previous guidelines [9]. This dramatic change generated a medical concern for hypothetical potential global impact. According to these new lipid goals, the proportion of subjects that reach the targets could be extremely low with the usual therapies. In that sense, our findings showed that many patients were subtreated and, consequently, lipid goals were poorly achieved. Less than 20% of diabetics classified as high or very high risk achieved the recommended LDL-C goal. Similar findings were observed when analyzing non-HDL-C.

The simulation model using adequate statin treatment showed that we could increase the proportion of very high risk diabetes patients who achieve the LDL-C target of <55 mg/dL from 16.4% to 35.5%. The increase was also observed in subjects with high risk (LDL-C of <70 mg/dL, from 18.8% to 56.8%) and moderate risk (LDL-C of <100 mg/dL, from 30.8% to 100%). Likewise, the simulation model using adequate treatment with statins and ezetimibe further increased the percentage of patients who reached the LDL-C goal. In this context, 51.8% and 73.2% of patients with high or very high risk achieved the recommended objective, respectively. Similar results were observed when analyzing non-HDL-C targets in both simulations.

There have been simulation models developed in Europe and the United States that evaluated patients with coronary heart disease [10,23]. Unlike these studies, our work exclusively evaluated patients with type 2 diabetes in Argentina.

Less than two years ago, European recommendations suggested considered “thresholds” of treatment to administer new drugs such as PCSK9 inhibitors [24]. These recommendations were based on the concept that the introduction of innovative therapeutic agents for the treatment of chronic disease states in large patient populations has important health economic implications. Patient groups at very high cardiovascular risk are likely to be a priority for this treatment. Likewise, the recent American College of Cardiology/American Heart Association clinical guidelines recognize the importance of considering economic value in making recommendations [2]. All models project higher lifetime costs from use of PCSK9 inhibitors because the cost will exceed any savings from prevention of cardiovascular events. To be cost effective by conventional standards, the cost of PCSK9 inhibitors will have to be reduced on the order of 70–85% in the United States. At any given price, the economic value of PCSK9 inhibitors will be improved by restricting their use to patients at very high risk of cardiovascular events.

It is imperative that better treatment strategies and methods be adopted to enhance diabetes control and reduce long-term complications of the disease. However, according to the findings of our work in an ideal setting, about half of very high risk patients would be candidates for PCSK9 inhibitors according to the new European guidelines. The economic consequences could be important and should be analyzed in the context of the advent of other expensive antidiabetic drugs, such as glucagon-like peptide-1 (GLP-1) receptor agonists or sodium–glucose cotransporter-2 (SGLT2) inhibitors [25,26]. According to the recently published guidelines on diabetes, prediabetes, and cardiovascular disease, patients with high or very high cardiovascular risk with glycated hemoglobin (HbA1c) greater than 7.0% (53 mmol/mol) should receive these drug groups. In our series, approximately 40% of patients meet these requirements. This number, associated with the percentage of PCSK9 inhibitor candidate patients, gives greater relevance to the above-described economic considerations.

This study had several limitations. First, it was cross-sectional with a moderate number of patients. Second, all participants were enrolled in cardiovascular prevention outpatient clinics of cardiology centers, which may have introduced selection bias. Third, we did not have enough samples to analyze other lipid markers such as apolipoprotein B. Finally, the number of patients classified as moderate risk was very limited.

## 5. Conclusions

The difficulty of achieving lipid goals in patients with type 2 diabetes was magnified by the recommendations of the recent European guidelines. This finding underlines the importance of initiatives to establish a more aggressive lipid management strategy. However, even under ideal treatment conditions, the proportion of subjects who should receive new and expensive drugs for the management of dyslipidemia would be considerable.

## Figures and Tables

**Table 1:** Characteristics of the population.

Continuous Variables *	N = 528
Age, years	62.1 ± 12.7
Diabetes time, years	5.5 (3.0–12.0)
Body mass index, kg/m^2^	31.4 ± 5.6
Creatinine, mg/dL	1.0 ± 0.4
Blood glucose, mg/dL	129.2 (110.5−151.5)
Glycated hemoglobin (HbA1c), % (mmol/mol)	7.1 (54) ±1.3
Total cholesterol, mg/dL	171.8 ± 43.4
LDL-C, mg/dL	95.8 ± 38.5
HDL-C, mg/dL	43.9 ± 12.4
Triglycerides, mg/dL	139.0 (100.0–189.0)
Non-HDL-C, mg/dL	127.9 ± 41.7
Systolic blood pressure, mmHg	128.7 ± 14.1
Diastolic blood pressure, mmHg	77.9 ± 10.2
Waist circumference, cm	106.0 ± 13.7
Categorical variables, %	
Male gender	64.0
Current smoking	10.0
Hypertension	70.3
Family history of early cardiovascular disease	11.2
Retinopathy	4.4
Neuropathy	5.7
Microalbuminuria	27.6
Obesity	55.7
Secondary prevention	37.3

* Values are expressed as mean ± standard deviation or median (interquartile range).

**Table 2:** Pharmacological treatment of the population (*n* = 528).

Treatment, %	*n* (%)
Aspirin	196 (37.1)
ACE inhibitors/angiotensin antagonists	336 (63.6)
β-adrenergic blockers	194 (36.7)
Calcium channel blockers	103 (19.5)
Diuretics	110 (20.8)
Biguanides (Metformin)	416 (78.8)
Sulfonylureas	53 (10.1)
Thiazolidinediones	11 (2.1)
Dipeptidyl peptidase-4 inhibitors	133 (25.2)
Glucagon-like peptide-1 receptor agonists	38 (7.2)
Sodium–glucose cotransporter-2 inhibitors	44 (8.3)
Atorvastatin	127 (24.1)
5 mg/day	2 (1.5)
10 mg/day	62 (47.7)
20 mg/day	42 (32.3)
40 mg/day	20 (15.4)
80 mg/day	4 (3.1)
Rosuvastatin	183 (34.6)
5 mg/day	30 (16.4)
10 mg/day	95 (51.9)
20 mg/day	53 (29.0)
40 mg/day	5 (2.7)
Simvastatin	20 (3.8)
10 mg/day	9 (47.4)
20 mg/day	10 (52.6)
Fluvastatin 80 mg/day	3 (0.6)
Ezetimibe	50 (9.5)
Fibrates	56 (10.6)
Omega 3	20 (3.8)

**Table 3:** Proportion of patients that achieved the lipid goals recommended by the 2019 European Guidelines on Diabetes according to the risk categories.

Lipid Goals	Moderate Risk *n* = 13 *n* (%)	High Risk *n* = 229 *n* (%)	Very High Risk *n* = 286 *n* (%)
LDL-C < 55 mg/dL			47 (16.4)
Non-HDL-C < 85 mg/dL			56 (19.6)
LDL-C < 70 mg/dL		43(18.8)	
Non-HDL-C < 100 mg/dL		42 (18.3)	
LDL-C < 100 mg/dL	4 (30.8%)		
Non-HDL-C < 130 mg/dL	6 (46.2%)		

**Table 4:** Use of the different statin schemes according to population risk.

Statin Doses	Moderate Risk *n* = 13 *n* (%)	High Risk *n* = 229 *n* (%)	Very High Risk *n* = 286 *n* (%)
High intensity	0	25 (10.9)	61 (21.3)
Moderate intensity	2 (15.4)	93 (40.6)	146 (51.1)
Low intensity	0	4 (1.8)	5 (1.7)
Without statins	11 (84.6)	107 (46.7)	74 (25.9)

**Table 5:** Proportion of patients that achieved lipid goals in the simulation analysis (ideal scenario where everyone received appropriate doses of statins ± ezetimibe).

Lipid Goals	Moderate Risk *n* = 13 *n* (%)	High Risk *n* = 229 *n* (%)	Very High Risk *n* = 286 *n* (%)
Adding statins at appropriate doses			
LDL-C < 55 mg/dL			100 (35.5)
Non-HDL-C < 85 mg/dL			103 (36.0)
LDL-C < 70 mg/dL		130 (56.8)	
Non-HDL-C < 100 mg/dL		132 (57.2)	
LDL-C < 100 mg/dL	13 (100)		
Non-HDL-C < 130 mg/dL	13 (100)		
Adding ezetimibe			
LDL-C < 55 mg/dL			129 (45.1)
Non-HDL-C < 85 mg/dL			148 (51.8)
LDL-C < 70 mg/dL		156 (68.1)	
Non-HDL-C < 100mg/dL		168 (73.2)	
LDL-C < 100 mg/dL	not applicable		
Non-HDL-C < 130 mg/dL	not applicable		

## References

[1] Mihaylova B., Emberson J., Blackwell L., Keech A., Simes J., Barnes E.H., Voysey M., Gray A., Collins R., Cholesterol Treatment Trialists’ (CTT) Collaborators (2012). The effects of lowering LDL cholesterol with statin therapy in people at low risk of vascular disease: Meta-analysis of individual data from 27 randomised trials. Lancet.

[2] Grundy S.M., Stone N.J., Bailey A.L., Beam C., Birtcher K.K., Blumenthal R.S., Braun L.T., Ferranti S., Faiella-Tommasino J., Forman D.E. (2018). 2018 AHA/ACC/AACVPR/AAPA/ABC/ACPM/ADA/AGS/AphA/ASPC/NLA/PCNA Guideline on the Management of Blood Cholesterol: A Report of the American College of Cardiology/American Heart Association Task Force on Clinical Practice Guidelines. J. Am. Coll. Cardiol..

[3] de Cardiología S.A., Normas Á.D.C.Y., Sociedad Argentina de Cardiología (2018). Uso apropiado de estatinas en Argentina. Documento de Posición. Rev. Arg. Cardiol..

[4] Breuker C., Clement F., Mura T., Macioce V., Castet-Nicolas A., Audurier Y., Boegner C., Morcrette E., Jalabert A., Villiet M. (2018). Non-achievement of LDL-cholesterol targets in patients with diabetes at very-high cardiovascular risk receiving statin treatment: Incidence and risk factors. Int. J. Cardiol..

[5] Gyberg V., De Bacquer D., De Backer G., Jennings C., Kotseva K., Mellbin L., Schnell O., Tuomilehto J., Wood D., Rydén L. (2015). Patients with coronary artery disease and diabetes need improved management: A report from the EUROASPIRE IV survey: A registry from the EuroObservational Research Programme of the European Society of Cardiology. Cardiovasc. Diabetol..

[6] Giugliano R.P., Cannon C.P., Blazing M.A., Nicolau J.C., Corbalán R., Špinar J., Park J.-G., White J.A., Bohula E.A., Braunwald E. (2018). Benefit of Adding Ezetimibe to Statin Therapy on Cardiovascular Outcomes and Safety in Patients With Versus Without Diabetes Mellitus. Circulation.

[7] Sabatine M.S., Leiter L.A., Wiviott S.D., Giugliano R.P., Deedwania P., De Ferrari G.M., Murphy S.A., Kuder J.F., Gouni-Berthold I., Lewis B.S. (2017). Cardiovascular safety and efficacy of the PCSK9 inhibitor evolocumab in patients with and without diabetes and the effect of evolocumab on glycaemia and risk of new-onset diabetes: A prespecified analysis of the FOURIER randomised controlled trial. Lancet Diabetes Endocrinol..

[8] Ray K.K., Colhoun H.M., Szarek M., Baccara-Dinet M., Bhatt D.L., Bittner V.A., Budaj A.J., Diaz R., Goodman S.G., Hanotin C. (2019). Effects of alirocumab on cardiovascular and metabolic outcomes after acute coronary syndrome in patients with or without diabetes: A prespecified analysis of the ODYSSEY OUTCOMES randomised controlled trial. Lancet Diabetes Endocrinol..

[9] Cosentino F., Grant P.J., Aboyans V., Bailey C.J., Ceriello A., Delgado V., Federici M., Filippatos G., Grobbee D.E., Hansen T.B. (2019). 2019 ESC Guidelines on diabetes, pre-diabetes, and cardiovascular diseases developed in collaboration with the EASD. Eur. Heart J..

[10] Gencer B., Koskinas K.C., Räber L., Karagiannis A., Nanchen D., Auer R., Carballo D., Carballo S., Klingenberg R., Heg D. (2017). Eligibility for PCSK9 Inhibitors According to American College of Cardiology (ACC) and European Society of Cardiology/European Atherosclerosis Society (ESC/EAS) Guidelines After Acute Coronary Syndromes. J. Am. Heart Assoc..

[11] Taskinen M.-R., Borén J. (2015). New insights into the pathophysiology of dyslipidemia in type 2 diabetes. Atherosclerosis.

[12] Comaschi M., Coscelli C., Cucinotta M., Malini P., Manzato E., Nicolucci A. (2005). Cardiovascular risk factors and metabolic control in type 2 diabetic subjects attending outpatient clinics in Italy: The SFIDA (survey of risk factors in Italian diabetic subjects by AMD) study. Nutr. Metab. Cardiovasc. Dis..

[13] März W., Dippel F.-W., Theobald K., Gorcyca K., Iorga Ş.R., Ansell D. (2018). Utilization of lipid-modifying therapy and low-density lipoprotein cholesterol goal attainment in patients at high and very-high cardiovascular risk: Real-world evidence from Germany. Atherosclerosis.

[14] Malik S., López V., Chen R., Wu W., Wong N.D. (2007). Undertreatment of cardiovascular risk factors among persons with diabetes in the United States. Diabetes Res. Clin. Pr..

[15] Shi Q., Liu S., Krousel-Wood M., Shao H., Fonseca V., Shi L. (2018). Long-term outcomes associated with triple-goal achievement in patients with type 2 diabetes mellitus (T2DM). Diabetes Res. Clin. Pract..

[16] Zhang X., Ji L., Ran X., Su B., Ji Q., Hu D. (2017). Gender Disparities in Lipid Goal Attainment among Type 2 Diabetes Outpatients with Coronary Heart Disease: Results from the CCMR-3B Study. Sci. Rep..

[17] Al-Zakwani I., Al-Mahruqi F., Al-Rasadi K., Shehab A., Al Mahmeed W., Arafah M., Al-Hinai A.T., Al Tamimi O., Al Awadhi M., Santos R.D. (2018). Sex disparity in the management and outcomes of dyslipidemia of diabetic patients in the Arabian Gulf: Findings from the CEPHEUS study. Lipids Health Dis..

[18] Moreno-Arellano S., De-Mendoza J.D.-, Santi-Cano M. (2018). Sex disparity persists in the prevention of cardiovascular disease in women on statin therapy compared to that in men. Nutr. Metab. Cardiovasc. Dis..

[19] Lin I., Sung J., Sanchez R.J., Mallya U.G., Friedman M., Panaccio M., Koren A., Neumann P., Menzin J. (2016). Patterns of Statin Use in a Real-World Population of Patients at High Cardiovascular Risk. J. Manag. Care Spéc. Pharm..

[20] Ziemer D.C., Miller C.D., Rhee M.K., Doyle J.P., Watkins C., Cook C.B., Gallina D.L., El-Kebbi I.M., Barnes C.S., Dunbar V.G. (2005). Clinical Inertia Contributes to Poor Diabetes Control in a Primary Care Setting. Diabetes Educ..

[21] García Díaz E., Ramírez Medina D., Morera Porras Ó.M., Cabrera Mateos J.L. (2019). Determinants of inertia with lipid-lowering treatment in patients with type 2 diabetes mellitus. Endocrinol. Diabetes Nutr..

[22] Masson W., Lobo M., Manente D., Vitagliano L., Rostán M., Siniawski D., Huerín M., Giorgi M. (2014). Respuesta a las estatinas en prevención cardiovascular. Evaluación de los hiporrespondedores. Rev. Argent. Cardiol..

[23] Rallidis L.S., Triantafyllis A.S., Iliodromitis E. (2018). Eligibility for treatment with PCSK9 inhibitors among patients with stable coronary artery disease presumed to be on maximum hypolipidaemic therapy. Hell. J. Cardiol..

[24] Landmesser U., Chapman M.J., Stock J.K., Amarenco P., Belch J.J.F., Borén J., Farnier M., Ference B.A., Gielen S., Graham I. (2017). 2017 Update of ESC/EAS Task Force on practical clinical guidance for proprotein convertase subtilisin/kexin type 9 inhibition in patients with atherosclerotic cardiovascular disease or in familial hypercholesterolaemia. Eur. Heart J..

[25] McEwen L.N., Casagrande S.S., Kuo S., Herman W.H. (2017). Why Are Diabetes Medications So Expensive and What Can Be Done to Control Their Cost?. Curr. Diabetes Rep..

[26] Soppi A., Heino P., Kurko T., Maljanen T., Saastamoinen L., Aaltonen K. (2018). Growth of diabetes drug expenditure decomposed-A nationwide analysis. Health Policy.

